# Leptospirosis in Mexico: Epidemiology and Potential Distribution of Human Cases

**DOI:** 10.1371/journal.pone.0133720

**Published:** 2015-07-24

**Authors:** Sokani Sánchez-Montes, Deborah V. Espinosa-Martínez, César A. Ríos-Muñoz, Miriam Berzunza-Cruz, Ingeborg Becker

**Affiliations:** 1 Unidad de Investigación en Medicina Experimental, Facultad de Medicina, Universidad Nacional Autónoma de México, Mexico City, Mexico; 2 Centro de Medicina Tropical, Facultad de Medicina, Universidad Nacional Autónoma de México, Mexico City, Mexico; 3 Museo de Zoología “Alfonso L. Herrera”, Departamento de Biología Evolutiva, Facultad de Ciencias, Universidad Nacional Autónoma de México, Mexico City, Mexico; University of Toledo School of Medicine, UNITED STATES

## Abstract

**Background:**

Leptospirosis is widespread in Mexico, yet the potential distribution and risk of the disease remain unknown.

**Methodology/Principal Findings:**

We analysed morbidity and mortality according to age and gender based on three sources of data reported by the Ministry of Health and the National Institute of Geography and Statics of Mexico, for the decade 2000–2010. A total of 1,547 cases were reported in 27 states, the majority of which were registered during the rainy season, and the most affected age group was 25–44 years old. Although leptospirosis has been reported as an occupational disease of males, analysis of morbidity in Mexico showed no male preference. A total number of 198 deaths were registered in 21 states, mainly in urban settings. Mortality was higher in males (61.1%) as compared to females (38.9%), and the case fatality ratio was also increased in males. The overall case fatality ratio in Mexico was elevated (12.8%), as compared to other countries. We additionally determined the potential disease distribution by examining the spatial epidemiology combined with spatial modeling using ecological niche modeling techniques. We identified regions where leptospirosis could be present and created a potential distribution map using bioclimatic variables derived from temperature and precipitation. Our data show that the distribution of the cases was more related to temperature (75%) than to precipitation variables. Ecological niche modeling showed predictive areas that were widely distributed in central and southern Mexico, excluding areas characterized by extreme climates.

**Conclusions/Significance:**

In conclusion, an epidemiological surveillance of leptospirosis is recommended in Mexico, since 55.7% of the country has environmental conditions fulfilling the criteria that favor the presence of the disease.

## Introduction

Leptospirosis is a zoonotic disease considered one of the most widely disseminated and prevalent in the wild, which can be transmitted by direct or indirect contact with urine of infected animals [1–5]. Humans are accidental hosts in the natural life cycle of the bacteria, and can show a wide range of symptoms such as febrile illness, headache, prostration, severe myalgia, uveitis (some of which may be mistaken with other infectious diseases like dengue, flu, hantavirus and rickettsiosis) [3], even renal failure and haemorrhagic manifestations also known as Weil’s disease. However, some infected people never show symptoms, having only a subclinical infection [1,2,4].

In Mexico, the first report of leptospirosis was made in patients from Merida, Yucatan, which had originally been diagnosed with yellow fever, yet the isolation of the bacteria proved leptospirosis [6]. Thereafter, a number of papers published between 1920 and 1990 reported the prevalence of the disease mostly in the southeast part of the country [7–10], yet little is known of the prevalence in the entire nation [11–13]. A report on the annual incidence of leptospirosis (one case per million inhabitants) done in 2007 showed that the majority of cases focalized in the state of Veracruz [14]. A more recent epidemiological overview of leptospirosis in Mexico between 2000–2010 showed a more widespread disease distribution [15].

Leptospirosis has been included in the group of re-emerging infectious diseases [16] and neglected zoonoses [17]. Annually, an estimated of 300,000–500,000 new acute cases emerge worldwide [18], with outbreaks occurring in several Latin-American countries, including Mexico. This calls for an analysis of epidemiological data through spatial epidemiology, which permits a description and analysis of geographical index health data with regard to demographic and environmental risk factors [19]. Epidemiological information can be accompanied by other techniques, such as ecological niche modeling. The combination of both sources of information can bring new perspectives to the analysis of infectious diseases [20–25], given that it is possible to characterize the potential geographic distribution based on environmental parameters [22, 23, 26].

Since leptospirosis remains a neglected disease and only scarce information exists on its impact on public health, our main objective was to identify the regions where leptospirosis could be present in Mexico, based on epidemiological data reported during the period 2000–2010, and also based on the same data, create a potential distribution map to identify the geographic distribution of the environmental variables associated to the presence of the disease. The combination of both sources of information permits the identification of geographical risk areas for the disease in Mexico and aids the design of preventive actions to limit the spread of the disease.

## Materials and Methods

### Epidemiological Data

We analysed morbidity and mortality data of leptospirosis in Mexico during the decade 2000–2010. The present analysis was done with three sources of data published by the Health Ministry and the National Institute of Geography and Statics of Mexico, based on the guidelines of *Norma Oficial Mexicana PROY-NOM-017-SSA2-2012 (para la vigilancia epidemiológica que establece la obligatoriedad y procedimientos generales de vigilancia de casos de Leptospirosis)*. The data do not include names or personal identification data of individual patients. This study was approved by the Ethics and Research Committee of the Medical Faculty of the UNAM (Universidad Nacional Autónoma de México) (FMED/CI/JMO/004/2012). Incidence data were divided into confirmed cases (patients that presented suggestive symptomatology of the disease and antibody titers of 1:800, including confirmation with a second sample in which the titers increased four times the initial value) and probable cases (patients with suggestive symptomatology of the disease and positive leptospira microagglutination or ELISA tests), according to the “Mexican Official Norm NOM-029-SSA2-1999, for the epidemiological surveillance, prevention and control of Leptospirosis in humans” [Norma Oficial Mexicana NOM-029-SSA2-1999, para la vigilancia epidemiológica, prevención y control de la Leptospirosis en el humano] [27]. We obtained data of: 1) confirmed cases from the database “Morbidity Yearbook during the period 1995–2010” [Anuarios de Morbilidad durante el periodo 1995–2010] and 2) for probable cases from the “Unique Information System for Epidemiological Surveillance [Sistema Único de Vigilancia Epidemiológica] (SUIVE)”, both generated by Ministry of Health of Mexico [Secretaría de Salud] [28]. The last source, 3) included data on deceased leptospirosis cases that were obtained from the database “Mortality Statistics” [Estadísticas de Mortalidad] of the National Institute of Geography and Statics of Mexico [Instituto Nacional de Estadística y Geografía] [29], that grouped the cases according to the International Statistical Classification of Diseases and Related Health Problems (ICD-10) [30]. Additionally, population statistics were obtained from the database “Basic Demographic Indicators 1990–2030” [Indicadores Demográficos Básicos 1990–2030] of the same institute [29].

It is important to clarify that data on specific serovars were not available for this study.

Analysis of cases was conducted according to age and gender of the patients and according to administrative entities (state) that notified the case by applying the Student's t-test for comparison of genders and the Kruskal-Wallis test (to compare mean values of patient ages and the state where patients had been reported) with post-hoc pairwise comparisons using Mann-Whitney U tests with Bonferroni correction. All tests were two tailed and *p* values <0.05 were considered significant. All statistical analyses were conducted using SPSS 20 [31, 32]. We obtained the annual incidence rates with the middle year population based on data from National Population Council of Mexico. We standardized rates with population of Mexico from 2005 with the use of Epidemiological Software Epidat 3.1 [33].

For mortality analysis, we conducted an analysis of specific mortality rates and case fatality ratio. In both cases the same multivariate analysis that had been used for morbidity data was included.

### Potential Distribution Map

#### Disease records

We obtained the geographic locations where the disease had been reported. Our records were divided in two groups defined as: validated cases [obtained from administrative entities that notified confirmed cases and deaths], and non-validated cases [including probable cases and deaths that were pooled together due to the unknown status of the patients and the possibility that they had not been infected in the administrative entity] [29]. The georeferentiation was based on data from “Catalogue of Keys from the Federal States, Municipalities and Localities” [Catálogo Único de Claves de Áreas Geoestadísticas Estatales, Municipales y Localidades] [34].

#### Ecological niche modeling approach and potential distribution

To create the potential distribution of the disease, we used a dataset of 19 bioclimatic variables, derived from temperature and precipitation, as shown in Table 1. Values of the bioclimatic variables were calculated based on the means of climatic records from 1910 to 2009 specifically for Mexico [35].

**Table 1:** Bioclimatic variables derived from temperature and precipitation.

Bio1	Annual mean temperature
Bio2	Mean diurnal range (mean monthly min. temp.—mean monthly max. temp.)
Bio3	Isothermality (Bio2/Bio7)
Bio4	Temperature seasonality (SD)
Bio5	Maximum temperature of the warmest month
Bio6	Minimum temperature of the coldest month
Bio7	Annual temperature range (Bio5-Bio6)
Bio8	Mean temperature of the wettest quarter
Bio9	Mean temperature of the driest quarter
Bio10	Mean temperature of the warmest quarter
Bio11	Mean temperature of the coldest quarter
Bio12	Annual precipitation
Bio13	Precipitation of the wettest month
Bio14	Precipitation of the driest month
Bio15	Precipitation seasonality (coefficient of variation)
Bio16	Precipitation of the wettest quarter
Bio17	Precipitation of the driest quarter
Bio18	Precipitation of warmest quarter
Bio19	Precipitation of coldest quarter

These layers correspond to climatic records from 1910 to 2009, which were specifically recovered for Mexico with a grid cell size of 0.01° x 0.01° (approx. 1 km2 x 1 km2) [35]. Although the inclusion of all 19 bioclimatic variables is prone to over-fitting [36], their use represents a conservative and a more reliable approach to estimate the potential distribution of the disease [37].

The potential distribution models were created using the Genetic Algorithm for Rule-set Production (GARP) implemented in the Desktop GARP software [38], which has proved to be a useful tool in understanding geographic and ecological distributions. GARP works in an iterative process through the formation of rules using inferential characteristics (atomic, range, negated range and logistic regression), the rules are evaluated and then considered to pass, or not, to a next generation. The process is stopped when a limited number of iterations are reached or when there is a limit of convergence and the new rules do not improve the model [39, 40].

A total of 100 replicates were generated using only the validated cases (those with information of prevalence and/or icterohemorrhagic leptospirosis). The data were split into training and testing points in a proportion of 70%–30%, respectively, in random seed to assure the randomness of the data to generate each replicate. Each replicate was made considering a limit of convergence of 0.01, which means that rules could not improve the result of the model in more than 1%, or when 1000 iterations were reached. The replica were validated by using a χ^2^ test, which considers the training and testing data, to evaluate the predictive capacities in each replicate used for binary predictions [41–43]. Then, from the 100 replicates, we selected the 10 best models based on minor omission errors and commission values close to the median [44]. These distribution models were summed to obtain only one final consensus model. This model was used to establish two thresholds of presence/absence of the disease, one for 90% of the best subset models [43] and other with 100% of the models.

Given that the non-validated data are not informative for epidemiological analyses, we decided to evaluate if it was possible to predict them using our final model. In order to prove that the association of the non-validated data was not the same as expected by chance, we used 100 sets of random points, where each set was formed with the same number of non-confirmed data.

To identify the values of the variables that could explain the potential distribution of the disease, we analysed the final model and the bioclimatic variables through a recursive partitioning analysis and a classification and regression tree, which are statistical procedures based on nonparametric regression methods [45]. Due to the large amount of pixels to be analysed in the final model, we resampled the geographical extent using a regular net of points separated every 5 km that represent a regular subsample of the presence/absence data in the model. The values of the bioclimatic layers were associated for each point in the net, and the classification and regression tree was constructed using JMP 9 [46]. The values considered on the tree were those present in the potential distribution model and that show a cumulative probability, based on the values of the variables considered in a nested form [45, 46].

The creation of the map was using the geographic information system QGIS 2.8.2-Wien (GNU General Public License) based on our presence point data and the results of the ecological niche modeling. We used the administrative areas of the World from the Global Administrative Areas 2.0 [47] and the Mexican administrative areas were provided by CONABIO [48] under licence CC BY-NC 2.5 MX (http://creativecommons.org/licenses/by-nc/2.5/mx).

## Results

### Epidemiological Overview

#### Distribution of cases and incidence

During the decade of 2000–2010, 1,547 confirmed cases were registered in 89 municipalities of 27 (84.4%) of the 32 states in Mexico. The state with highest number of cases was Veracruz with 377 (24.4%), followed by Tabasco 260 (16.8%), Sinaloa 129 (8.3%), Hidalgo 116 (7.5%) and Oaxaca 97 (6.3%). The states with lowest number of cases were Nayarit and Tlaxcala 3 (0.2%), Michoacan 2 (0.1%) and Aguascalientes 1 (0.1%). States that never reported cases were: Baja California, Durango, Guanajuato, Queretaro and Zacatecas (Table 2).

**Table 2:** Epidemiological overview of leptospirosis by states in Mexico, during the period 2000–2010.

State	Cases	Deaths	Incidence rate*	Specific mortality rate*	Case fatality ratio (%)
**Aguascalientes**	1	0	0.08	0	0
**Baja California**	0	0	0	0	0
**Baja California Sur**	5	0	0.8	0	0
**Campeche**	23	6	2.82	209.57	26.09
**Coahuila**	4	0	2.77	0	0
**Colima**	6	0	0.62	0	0
**Chiapas**	75	2	0.08	11.75	50
**Chihuahua**	4	1	0.18	5.78	16.67
**Distrito Federal**	80	44	0.92	70.82	55
**Durango**	0	0	0	0	0
**Guanajuato**	0	1	0	4.31	0
**Guerrero**	65	9	1.93	79.93	13.85
**Hidalgo**	116	0	4.39	0	0
**Jalisco**	10	2	0.14	5.76	20
**Mexico State**	47	6	0.31	12.32	12.77
**Michoacan**	2	1	0.05	5.03	50
**Morelos**	5	2	0.29	25.51	40
**Nayarit**	3	0	0.28	0	0
**Nuevo Leon**	4	1	0.09	5.02	25
**Oaxaca**	97	3	2.62	10.85	2.02
**Puebla**	16	3	0.28	7.11	12.5
**Queretaro**	0	0	0	0	0
**Quintana Roo**	16	2	1.22	32.78	6.25
**San Luis Potosi**	35	0	1.37	0	0
**Sinaloa**	129	65	4.68	585.31	51.94
**Sonora**	92	22	3.48	166.20	22.83
**Tabasco**	260	11	11.74	121.55	4.23
**Tamaulipas**	16	1	0.5	7.16	6.25
**Tlaxcala**	3	1	0.26	22.18	33.33
**Veracruz**	377	14	4.99	37.67	3.71
**Yucatan**	56	2	2.88	31.22	5.36
**Zacatecas**	0	0	0	0	0
**Total**	1547	198	-	-	-

*Rate per 100,000 inhabitants

A significant difference is observed when comparing the mean annual data of cases obtained in the states: Distrito Federal, Hidalgo and Veracruz (χ^2^ = 35.88, *p* = <0.001).

The number of cases by year ranged from 40 to 483, with a median of 141 annual cases. Incidence rate varied from (0.04) to (0.44) per 100,000 inhabitants. During the entire period, the number of cases increased 12 times, whereas the incidence rate rose 11 times.

**Fig 1:**
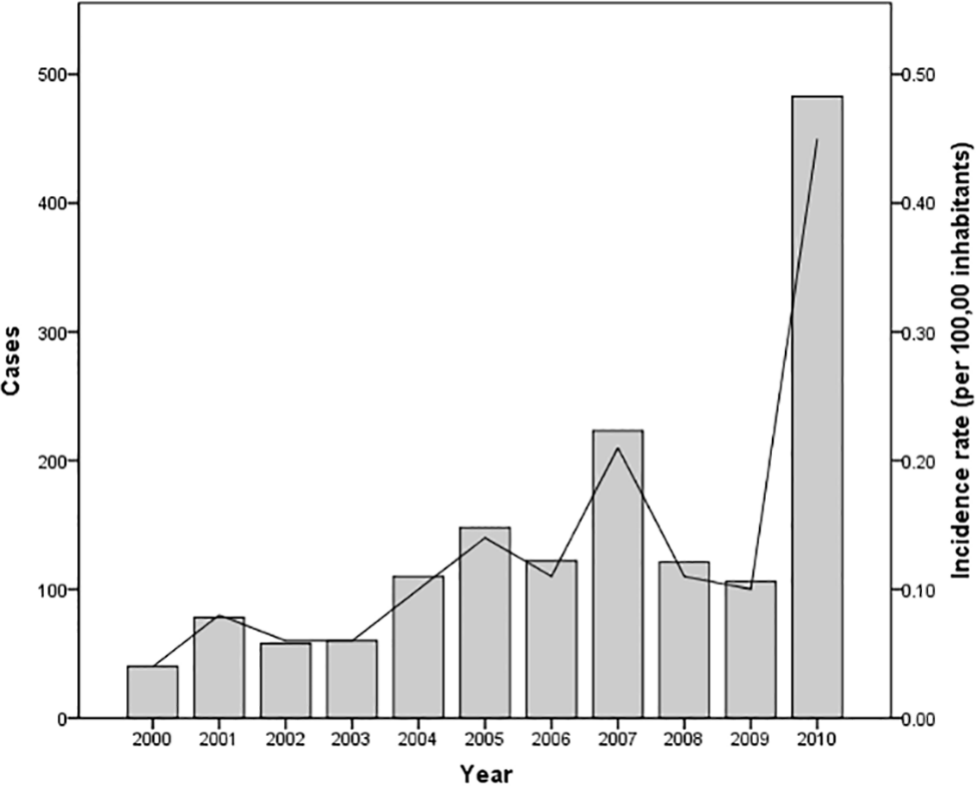
Distribution of cases and incidence rate of leptospirosis registered in Mexico during the period 2000–2010. Number of cases are represented by bars and the incidence rate is represented by the solid line.

We observed two peaks: one occurred in 2007 with 223 cases (an incidence rate of 0.21 per 100,000 inhabitants) and one in 2010 with 483 cases (incidence rate of 0.44 per 100,000 inhabitants) (Fig 1).

**Fig 2:**
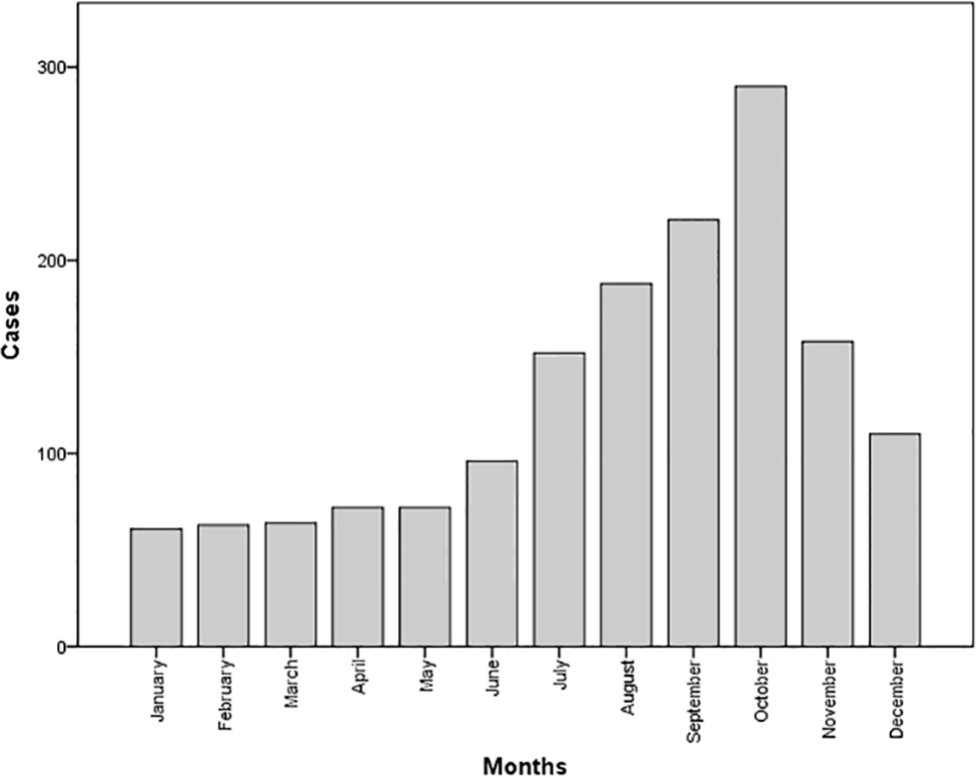
Monthly distribution of leptospirosis cases in Mexico during the period 2000–2010. Number of cases are represented by bars.

During the whole period 2000–2010, the majority of cases were reported during the rainy season (throughout the second semester of the year). A total of 1,121 cases (72.5%) were reported during July through December, with the increase in the registration beginning in June (with a mean number of 60 to 80 cases), reaching the highest number in October (with 279 cases), which represents an increase of 3.4 times, as compared to previous months (Fig 2).

#### Age and gender

We could not analyse the incidence according to gender for the entire period since the Mexican Ministry of Health began notifying the gender of the patients after 2002. However, the analysis done from 2003–2010 showed no significant differences between male and female cases, where 760 (55.4%) cases were male and 613 (44.6%) were female, and incidence rates between both groups showed no significant differences (Table 3).

**Table 3:** Cases and incidence rate of leptospirosis according to gender, during the period 2003–2010 in Mexico.

	General		Females			Males		
Year	Cases	%	Cases	%	Incidence rate*	Cases	%	Incidence rate*
**2003**	60	4.4	24	3.9	0.07	36	4.7	0.05
**2004**	110	8.0	61	10	0.1	49	6.4	0.12
**2005**	148	10.8	67	10.9	0.15	81	10.7	0.13
**2006**	122	8.9	52	8.5	0.13	70	9.2	0.1
**2007**	223	16.2	92	15.0	0.25	131	17.2	0.17
**2008**	121	8.8	54	8.8	0.13	67	8.8	0.1
**2009**	106	7.7	45	7.3	0.11	61	8.0	0.1
**2010**	483	35.2	218	35.6	0.50	265	34.9	0.39
**Total**	1373	100	613	100		760	100	

*Rates per 100,000 inhabitants. χ^2^ = 112.304, *p* = >0.005

**Fig 3:**
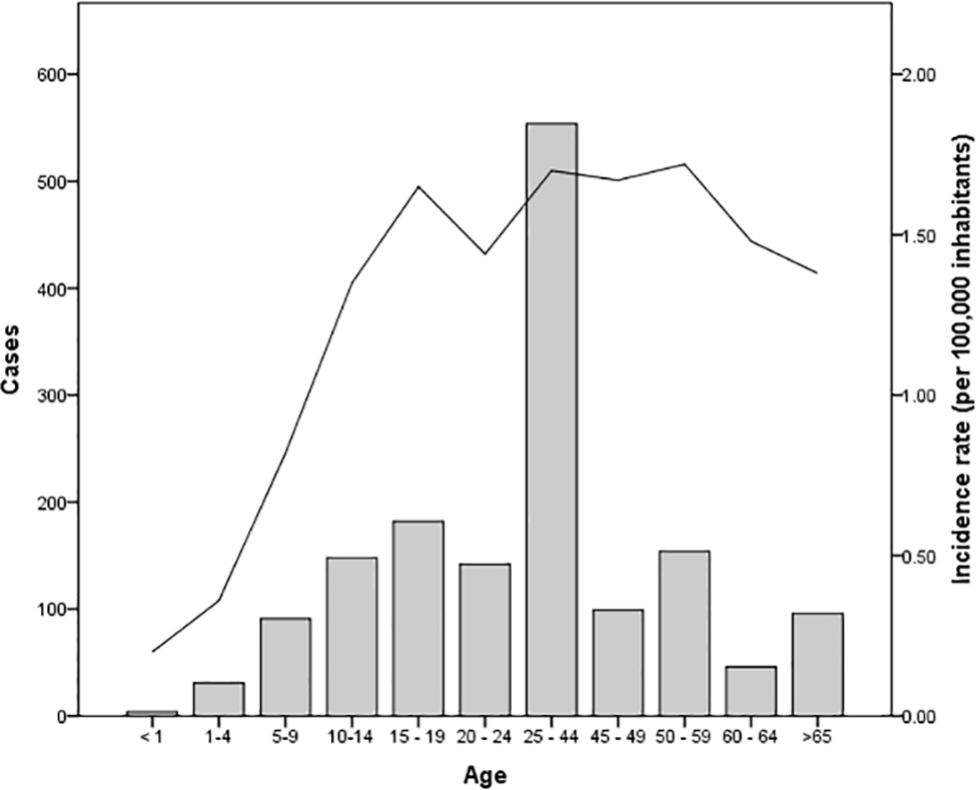
Cases and infection rates according to age. The bars represent the number of cases for each age group that was registered during the decade. The solid line represents the incidence rate per 100,000 inhabitants).

The age group showing the highest number of cases was 25–44 years with 554 cases (35.8%), followed by the age group of 15–19 with 182 cases (11.8%). The lowest number of cases corresponded to children aged less than one year, with only 4 cases. With regard to infection rates, the 50–59 year age group had the highest infection rate (1.72 per 100,000 inhabitants) as compared to children younger than one year of age, showing 0.20 per 100,000 inhabitants (Fig 3).

#### Specific Mortality and Case Fatality Ratio

A total of 198 confirmed deaths were registered in 21 states of Mexico during 2000–2010 (Table 2). The number of deaths differed significantly between the states (χ^2^ = 112.304, *p* = <0.001). States with high number of deaths include Sinaloa with 65, followed by Distrito Federal with 44. In contrast, Chihuahua, Guanajuato, Michoacan, Nuevo Leon, Tamaulipas and Tlaxcala notified only one case during the notification period. Interestingly, although Hidalgo exhibited a high incidence rate (4.39 per 100,000), this state reported no deaths (Table 2).

**Fig 4:**
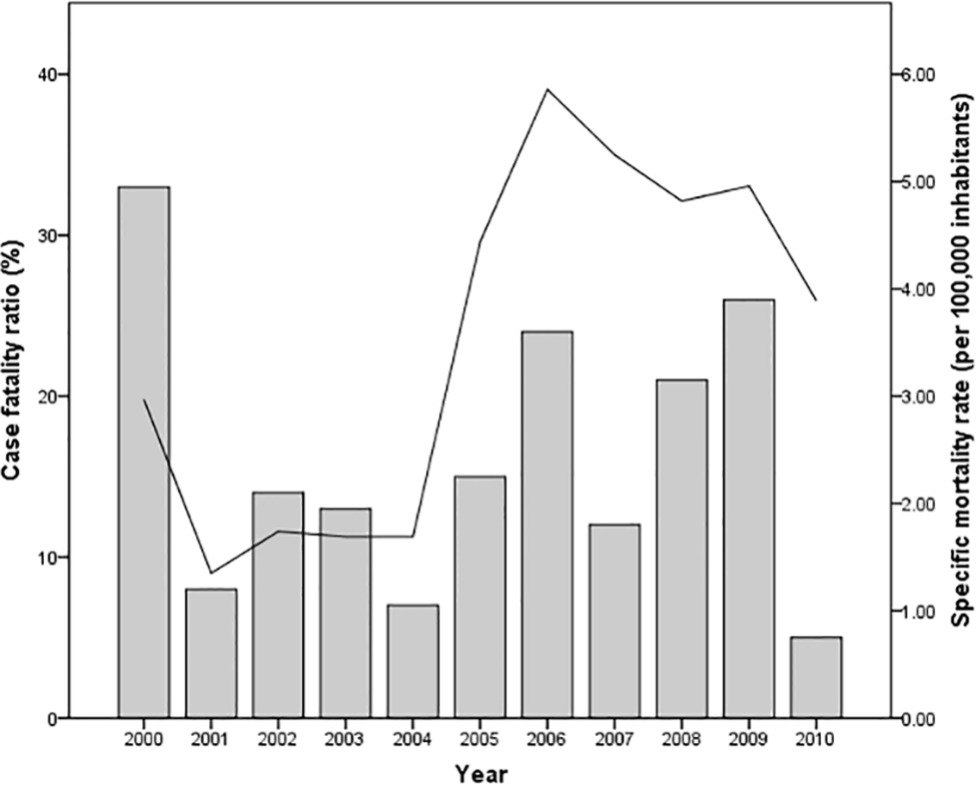
Distribution of leptospirosis case fatality ratio and specific mortality rate registered in Mexico during 2000–2010. The bars represent case fatality ratios (%) and solid line represents specific mortality rates (per 100,000 inhabitants) registered during the decade.

Throughout the decade, the specific mortality rate for leptospirosis varied between 1.25 and 5.86 per 100,000, which triplicated during 2004–2006. The case fatality ratio during this decade exhibited a mean value of 12.8% (198/1547), where the highest ratio was registered in 2000 with 33% (13/40). Although the ratio decreased to 11.4% during the following five years, a renewed temporal increase surged in 2006 showing a 24% (29/122) case fatality ratio, which diminished drastically in 2010 to 5% (23/483), reaching the lowest value of the decade (Fig 4).

We additionally obtained information on the type of locality (rural/urban) where the 198 deaths were reported. Significantly higher numbers of deaths were reported in urban areas with 161 cases (81.3% with a mean of 14.5 deaths per year) against 35 (17.7% with a mean of 3.5 deaths per year) reported in rural settings, *p* = 0.0005; only 2 cases (1.0%) were classified as unknown.

The analysis of the deceased patients according to gender, revealed higher numbers (121) in male (61.1%) in contrast to 77 female patients (38.9%), albeit the difference was not significant (*p* = 0.105). A significant difference in case fatality ratios was observed between genders, being 17.5% (107/613) for males and 8.4% (64/760) for female patients (*p* = 0.034).

**Fig 5:**
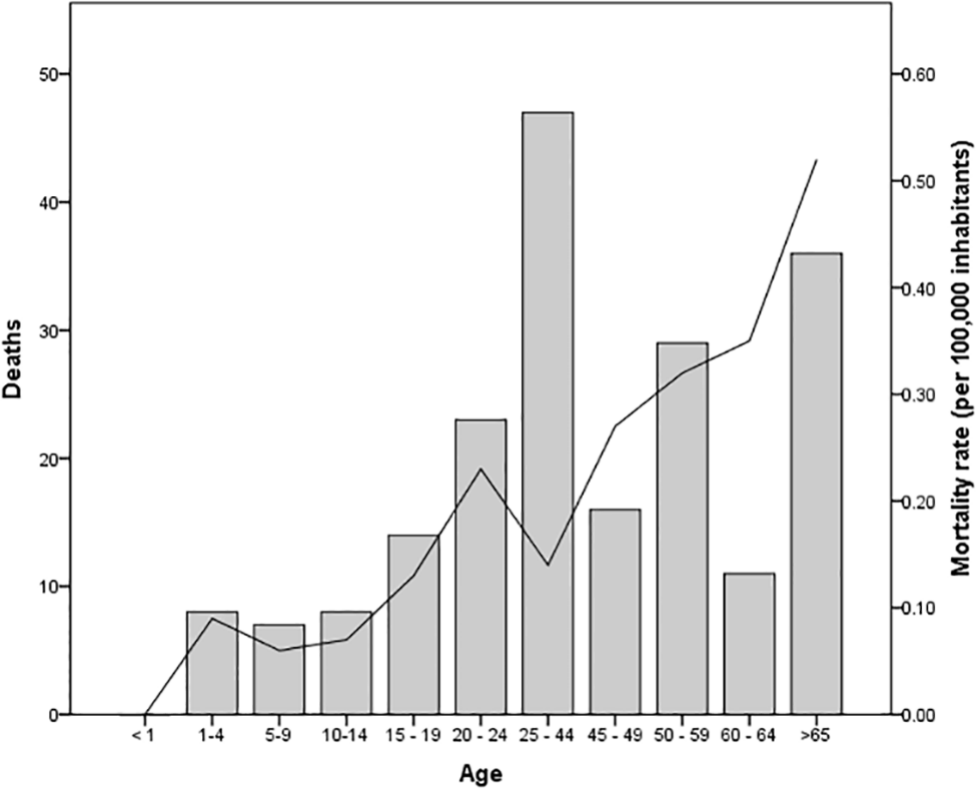
Specific mortality rates and case fatality ratio of leptospirosis according to age. The bars represent number of patient deaths and solid line represents specific mortality rates (per 100,000 inhabitants) registered during the decade.

There was a significant difference in the number of deaths from leptospirosis according to age (χ^2^ = 45.911, *p* = <0.001). The age group of 25–44 years showed 47 deaths (24%), followed by adults over age 65 with 36 deaths (18%) and adults 50–59 with 29 fatalities (15%). No deaths were recorded in children with less than one year of age, which differs significantly from the other age groups that report high numbers. The mortality rate increases with age, with the highest value recorded in the larger group of >65, followed by 60–64 years. Although the number of deaths is concentrated in the group of 25–44 years, the mortality rate decreases because it comprises a much wider group of people (Fig 5).

### Ecological Overview

#### Ecological niche modeling and potential distribution

**Fig 6:**
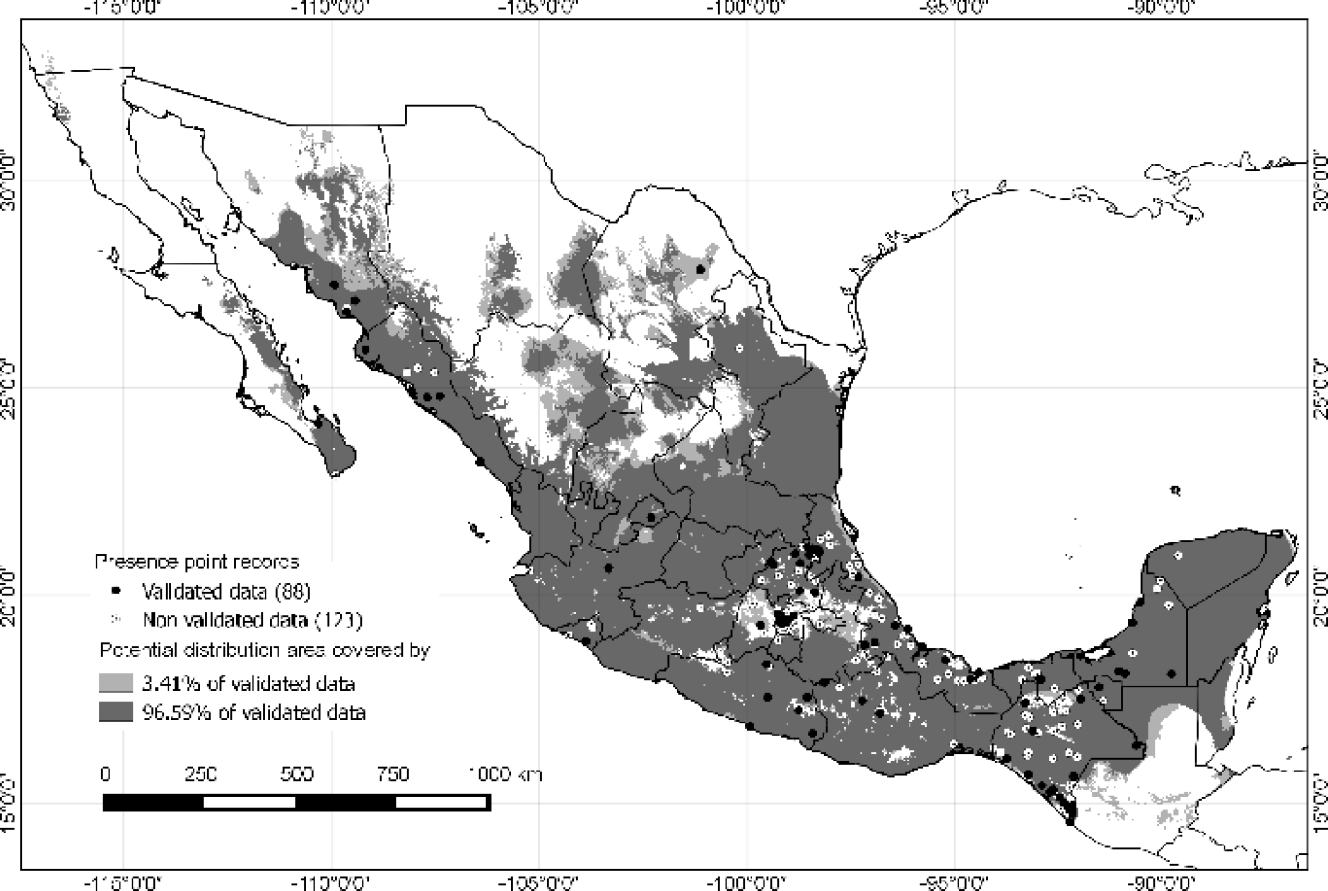
Map showing presence of cases and potential distribution of leptospirosis in Mexico. The potential distribution was created with ecological niche modeling using validated cases. Dark points show localities of validated data (confirmed leptospirosis cases and deaths), light points show non-validated data (probable cases and deaths that were pooled together due to the unknown status of the patients). Grey tones show the two thresholds used to determine the potential distribution of the disease. Dark grey shows the threshold of 96.59% of the best subsets. Light grey shows the remaining 3.41% of the validated data considered as possible errors.

We obtained a database with 88 spatially unique validated records, and 123 spatially unique non-validated data. Potential distribution was based on the validated data, showing wide predictive areas in the country, mainly in southern Mexico. There was no distribution in regions with extreme climates (extremely cold and dry) and no prediction was evidenced in highlands or in the north of Mexico (Fig 6).

Given the possibility of errors in the geo-references, we took two thresholds into account: the congruence of all best subset models that predicted all validated data (light and dark grey) and a threshold of 96.59% of the best subsets (only the dark grey area), which left out 3.41% of the validated data. The use of both thresholds showed the possible restrictiveness, if those records that did not coincide with all the best subsets were considered as errors (which corresponded to only 3.41% of the validated data). The restrictiveness is evidenced only in the north and in the highlands of central Mexico, and does not change the pattern previously described (Fig 6).

Despite the fact that non-validated data are not epidemiologically informative, we nonetheless used this model to test if leptospirosis cases could be predicted. The potential distribution map could help clarify if non-validated data could be compared with validated data. The wide potential distribution easily showed that non-validated data were correctly predicted.

The recursive partitioning analysis and the classification and regression tree were calculated for the predictive distribution with the threshold of 100%, which considers the area where all the best subset models coincide. Given the nested characteristics of the analyses, it was important to consider the order of the variables, since the first variables represent a prerequisite for the next variables. It was possible to identify that temperature variables were those that first explained the predictive distribution model, and were also the most informative. The first two temperature variables (Bio4 and Bio2, Table 4) explained up to 75% of the potential distribution. Precipitation variables, on the other hand, contributed once the temperature variables were already present and their contribution was lower (Table 4).

**Table 4:** Probabilities of presence for leptospirosis in Mexico according to the recursive partition analysis of bioclimatic variables and values.

Cumulative environmental variables		Cumulative probability	Contribution (%)
Bio4 Temperature seasonality (SD)	< 2.009632	0.6195	61.95
Bio2 Mean diurnal range (mean monthly min. temp.—mean monthly max. temp.)	< 16.84381	0.7504	13.09
Bio8 Mean temperature of the wettest quarter	≥ 15.60133	0.8115	6.11
Bio3 Isothermality (Bio2/Bio7)	≥ 0.4397839	0.8498	3.83
Bio3 Isothermality (Bio2/Bio7)	< 0.7535372	0.8811	3.13
Bio13 Precipitation of the wettest month	≥ 15.51667	0.9359	5.48
Bio11 Mean temperature of the coldest quarter	≥ 9.972473	0.9638	2.79
Bio2 Mean diurnal range (mean monthly min. temp.—mean monthly max. temp.)	≥ 9.190779	0.9774	1.36
Bio1 Annual mean temperature	< 27.42323	0.9833	0.59
Bio14 Precipitation of the driest month	< 18.87975	0.9875	0.42
Bio9 Mean temperature of the driest quarter	≥ 11.79974	0.9905	0.3
Bio18 Precipitation of warmest quarter	≥ 87.88699	09922	0.17

## Discussion

Here we show the first database compilation on leptospirosis in Mexico with an analysis of morbidity and mortality, obtained from three official sources [28, 29]. Since this disease is not officially notified and no official standards for notification exist, the actual numbers of patients remain unknown and the serovars of the *Leptospira* are not always reported in some of the states. Thus, the actual magnitude of the potential distribution and risk of the disease remain unknown. In an attempt to determine the potential distribution of the disease in Mexico, we considered all patients with leptospirosis as a group, irrespective of serovars. When we initiated this analysis, and based on previously published papers on leptospirosis as a water borne disease [1, 2, 4], we expected to find a close correlation between patients with leptospirosis and water. This might hold true for the outbreaks, but precise data of a possible more widespread distribution of the disease throughout the country and factors associated with the disease occurrence have not been analysed. We were interested in studying leptospirosis at a national level, seeking more detailed correlations between the disease distribution with bioclimatic variables derived from temperature and precipitation, in order to clarify which factors play a role in the establishment of the disease.

Leptospirosis had been previously reported as an occupational disease of males [49–51]. Yet our study shows that in Mexico there is no male preference in morbidity, although the case fatality ratio in male patients is significantly higher than in females, which is in accordance with a previous study in Portugal [52]. The overall case fatality ratio reported in Mexico (12.8%) is higher as compared to other countries such as Barbados 9.8%, [53], Morocco 9.5% [54], Germany 8% [55], Trinidad and Tobago 5.8% [56] and Hawaii, USA 0.5% [57]. This is possibly related to problems in the early diagnosis and opportune treatment, yet the underlying cause of the higher male fatality ratio needs to be investigated.

Our data additionally show that disease morbidity has increased during the last decade. This is possibly due to two reasons: in 2010 an active search for patients with dengue was established after several environmental contingencies had occurred (in this period there were a total of 33 tropical cyclones and two hurricanes Agatha and Igor) in the coastal areas of the Pacific and the Atlantic [58, 59]. The second reason for the heightened number of cases is possibly due to the fact that 17 state laboratories were established (certified for the diagnosis of leptospirosis) and assigned to the National Network of Public Health Laboratories [60], which increased the number of analysed samples. The reduction of patients deaths observed during 2010 is possibly related to a better and opportune early diagnosis enabling a timely treatment.

Within Mexico, the incidence rate and case fatality ratios reported in the different states vary importantly. Furthermore, not all the states that have a high incidence rate also report a large number of patient deaths. This is the case of Hidalgo, a state with the highest incidence rate, yet no patient deaths are reported (Table 2). This could be due to different serovars of the bacteria, which can differ widely in their virulence [2–4]. As most states do not report serovars, this finding calls for the urgent need to report the infecting ones in the affected areas. In addition to serovars, local diagnostic and management facilities must also be taken into account.

Our study also shows that leptospirosis related deaths are predominantly reported in urban areas. This contrasts with the rural distribution of disease morbidity [2, 3]. The cause of this apparent discrepancy can be due to the fact that severely ill patients are transferred to hospitals in major cities, where their decease is finally reported after an obligatory autopsy.

This finding calls for sanitary precautions that need to be attended by health care professionals to avoid infection and undesired spread of the disease.

Our current data open a new perspective of leptospirosis in the country, since it shows a wide scope of environmental factors associated with the disease distribution. Previous reports have only shown pinpoint cases [12], yet our map shows a geographic continuity making it plausible that the disease can have a much wider distribution than expected by sanitary authorities, using models generated for another infectious disease like Chagas disease [61, 62], Dengue [63] and Hantavirus [64]. The analysis of potential distribution based on ecological niche modeling, allows us to associate ecological and biogeographical factors of the pathogen [42, 65]. Even though other biotic factors can be involved in the dynamics of the leptospirosis, such as vectors or host species, these are not easy to represent in the potential distribution hypothesis, because the lack of information does not permit a precise assessment of their specific role, especially if we consider that new host species for leptospirosis are currently being reported in Mexico [66].

Furthermore, our analysis shows that non-validated data share the same ecological characteristics as validated data, and therefore these areas should be followed up closely since they are potentially equally exposed to the disease occurrence. A potential impact of our map is showing that states such as Durango, Queretaro and Zacatecas, that have no epidemiological report of patients, lie within a region likely of having leptospirosis. Thus, it could be possible that the disease is present and should be sought for in humans.

Our study is in accordance with the literature showing that the disease occurs mainly during the rainy season and that it affects predominantly adults [51, 67, 68]. Yet our bioclimatic analysis additionally shows that temperature plays a more determining role in the distribution of leptospirosis cases, as compared to precipitation variables. This finding helps explain the cases reported in desert areas, such as those reported in Israel [69]. Yet this does not hold true for leptospirosis outbreaks, since these are clearly associated with natural disasters such as floods and hurricanes. In Europe, the reported outbreaks were also associated with temperature, since all cases occurred during the hottest summer months [52, 70, 71].

An interesting phenomenon on geographical interference of disease distribution was evidenced in the Distrito Federal, where a high number of cases have been reported (Table 2). A mountain range passes through the southern part of the city forming a corridor that could be responsible for limiting the spread of the disease towards the southern part, which is confirmed by the lack of patients in this geographical region. This contrasts with the northern semi-desert areas where ecological conditions are permissive for leptospirosis and where patients have been reported.

In conclusion, our findings show that not only precipitation but also temperatures are predictors of the disease distribution. Based on our current analysis, an active epidemiological surveillance of leptospirosis is recommended in Mexico, since 55.7% of the country has ecological conditions fulfilling the criteria to harbour the disease. Clearly, leptospirosis is an emerging disease worldwide that can become a considerable public health problem in Mexico if left unattended.
